# Terrain Awareness Using a Tracked Skid-Steering Vehicle With Passive Independent Suspensions

**DOI:** 10.3389/frobt.2019.00046

**Published:** 2019-06-21

**Authors:** Rocco Galati, Giulio Reina

**Affiliations:** Department of Engineering for Innovation, University of Salento, Lecce, Italy

**Keywords:** mobile robotics, terrain recognition, tracked vehicles, skid-steering, articulated suspension system

## Abstract

This paper presents a novel approach for terrain characterization based on a tracked skid-steer vehicle with a passive independent suspensions system. A set of physics-based parameters is used to characterize the terrain properties: drive motor electrical currents, the equivalent track, the power spectral density for the vertical accelerations and motor currents. Based on this feature set, the system predicts the type of terrain that the robot traverses. A wide set of experimental results acquired on various surfaces are provided to verify the study in the field, proving its effectiveness for application in autonomous robots.

## 1. Introduction

During the last decade, several robotic solutions have been developed to support human workers in agricultural and industrial activities, i.e., spraying, mining, harvesting, good transportation, and plant monitoring with heavy duty operation. Moreover, the use of a large set of sensors like RGB cameras, lasers, GPS, and inertial sensors allow the robots to adapt their system to the environment by processing input data against a large set of data (Narvaez et al., 2017). Anyway, while most of the previous studies on off-road mobile robots focuses on obstacle detection (Schaefer et al., 2005), path planning (Elfes et al., 1999), and position estimation (Henson et al., 2008), not so much attention has been devoted to the interaction between the robot and the terrain and how this interaction affects the vehicle performance during normal operations. Surely, in most rough outdoor applications, the classification, and the characterization of the terrain is the key for robot autonomy and safety: the correct evaluation of the terrain features allows the vehicle to optimize its speed and drive torque and, particularly, to avoid hazardous conditions that can damage its locomotion system or endanger the vehicle itself. As a notable example, identifying terrain type is critical for the safety of planetary exploration rovers, such as the NASA/JPL rovers (Rothrock et al., 2016). The approaches in the literature used for terrain characterization usually require off-line processing and specific sensors and devices that can be expensive and complex to handle in rough environments (Ojeda et al., 2006). Examples of exteroceptive sensing can be found in Milella et al. (2015) where a combination of radar and monocular vision within a self-learning statistical framework was presented to classify agricultural terrain. A local descriptor obtained from 3D environment reconstruction was proposed in Bellone et al. (2018) for terrain unevenness estimation. Laser rangefinders and spectral imaging sensors were also proposed for ground identification, respectively, in Broten et al. (2012) and Jin et al. (2015).

Other researchers have investigated terrain classification methods using proprioceptive sensing. For example, acceleration-based terrain classification methods were introduced for planetary exploration rovers (Brooks and Iagnemma, 2005) and rough-terrain robots (DuPont et al., 2008). However, the vehicles adopted for the tests are based on wheels and they are typically equipped with no suspension systems (Masha et al., 2017; Reina et al., 2017a). This last aspect can be considered as a limiting factor since the roughness that it is possible to run against in terrains like ploughed and rocky soil or gravel can generate unintended mechanical stress to the robot frame and the sensors.

This study proposes a method for terrain characterization using a tracked skid-steering vehicle with passive suspensions and by defining a set of parameters that are based on the physical understanding of the mechanisms underlying the vehicle-terrain interaction, namely, the drive motor currents, the equivalent slip track and the power spectral density associated to the electrical currents and body vertical accelerations. The first two parameters are strictly connected to the power needed by the vehicle to face a specific terrain, i.e., sand generates a larger resistance to motion than asphalt; the equivalent slip track can be used to measure the amount of slippage associated to a skid-steering vehicle during a steering maneuver. An Extended Kalman Filter (EKF) is used to support a model-based estimator in order to provide an online estimation of the slip track; the filter uses as input the difference between the left and right tracks velocities obtained by using rotary encoders mounted on both tracks sprockets and the vehicle way rate measured by an inertial unit. The power spectral density (PSD) of the vertical acceleration describes the power in the signal as a function of frequency, per unit frequency (Li and Sandu, 2013). In our study, the robot vertical motion is controlled by the shock absorber mounted on each suspension arm.

Following the paper organization, section 2 illustrates the vehicle model used for this research. Section 3 investigates how the vehicle interacts with its supporting surface during straight and turning motion and it provides a description of the PSD method. Section 4 provides considerations and experimental results obtained on different surfaces by using an all-terrain tracked vehicle to validate the proposed approach. Section 5 concludes the paper.

## 2. Materials and Methods

### 2.1. The Hardware Architecture

**Figure 1:**
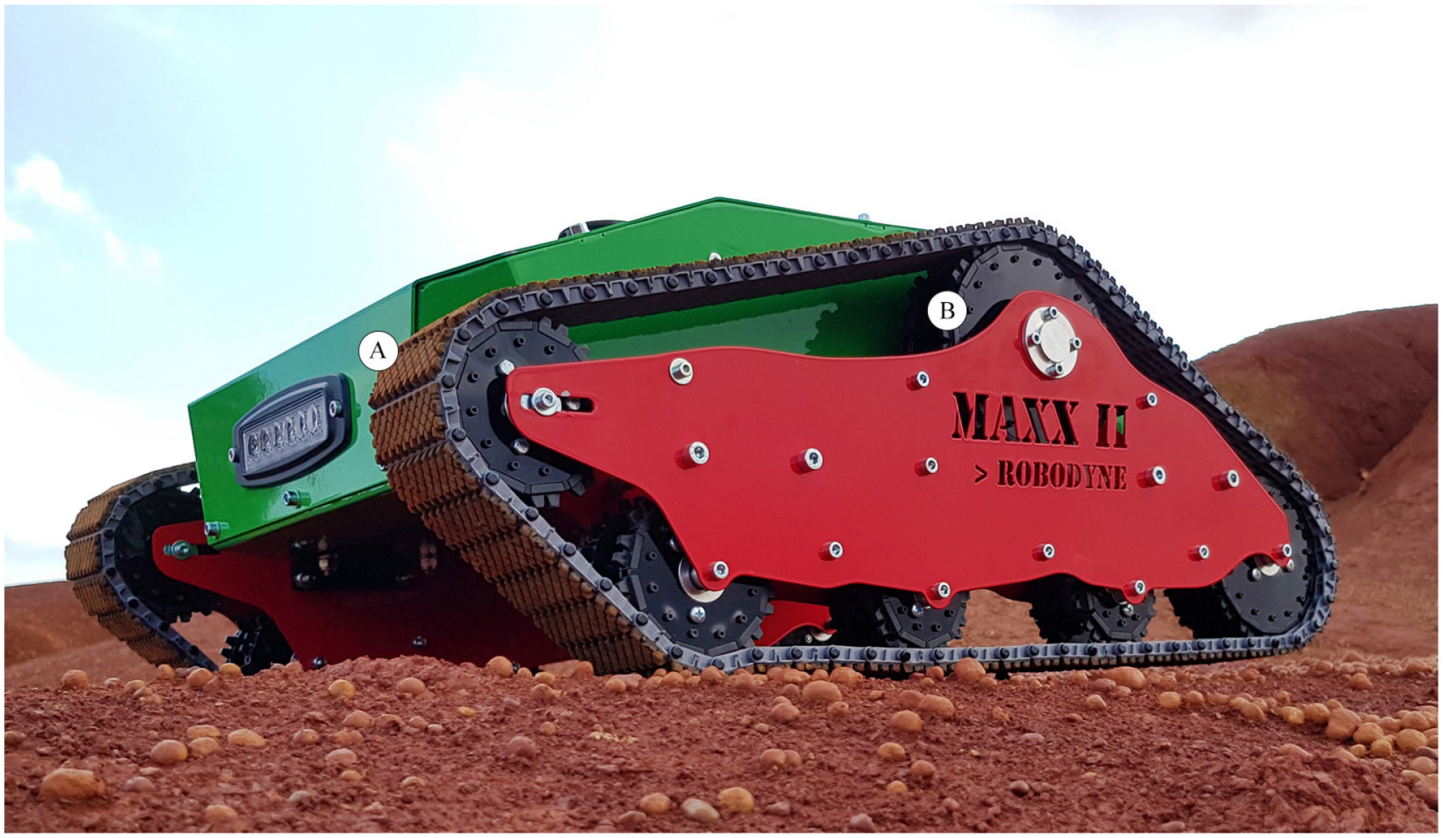
A first version of the vehicle “maXXII” used for this research work.

The vehicle used for this research work is a tracked skid-steering robot named “maXXII” that is under development at the University of Salento. It features passive suspensions as showed in Figure 1. The weight of the vehicle is *W* = 40 kg and its nominal track width is 0.95 m. Each track pad (A) has a width of about 0.18 m and a height of 0.16 m and it is composed by a continuous band of treads made of synthetic rubber for off-road use and reinforced with steel wires in order to provide a good traction on almost all the surfaces. Each undercarriage is based on a parallelogram design with an upper front wheel more advanced to help the vehicle to overcome obstacles and climb stairs. Each track sprocket (B) is driven by a 12V DC motor with a gearbox with a maximum output torque of 40 Nm and a maximum angular velocity of 70RPM for a total output power of about 400 W. The sensor set includes two optical encoders mounted on each gearbox shaft, two current sensors, a RTK GPS and an inertial measurement unit with 3-axis gyroscope, accelerometer and magnetometer for the orientation following the NED (North, East, Down) reference frame.

### 2.2. The Suspension System

**Figure 2:**
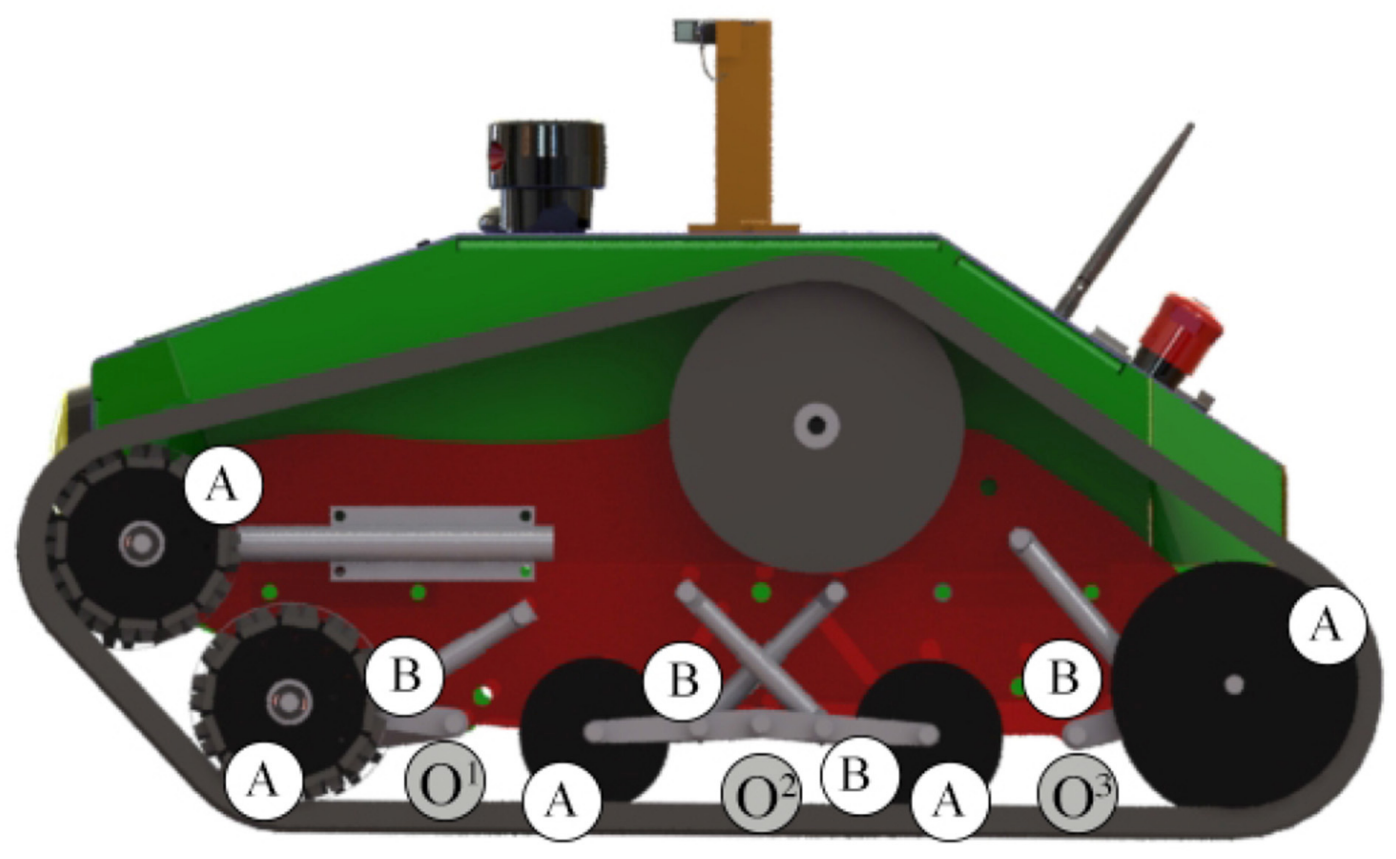
The passive suspension system used for each track made by four linkages and four shock absorbers.

**Figure 3:**
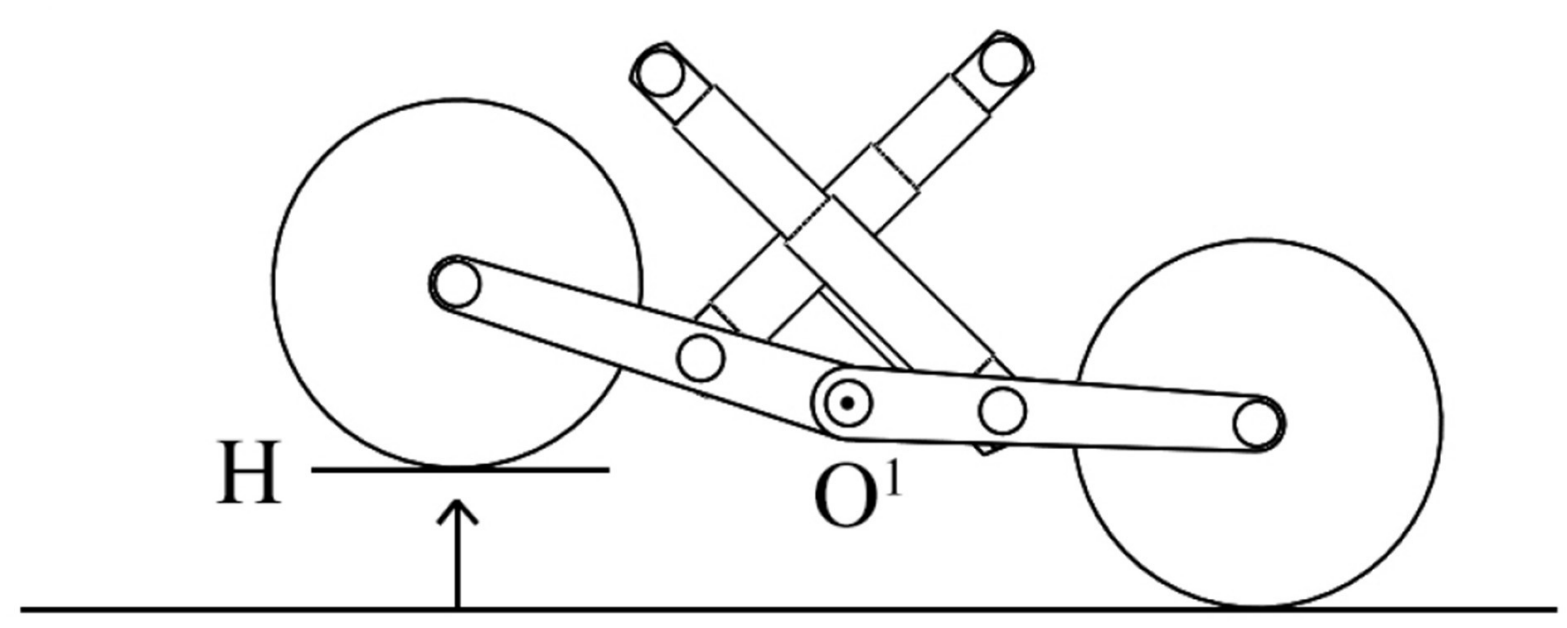
Single-arm suspension linkages with shock absorbers.

**Figure 4:**
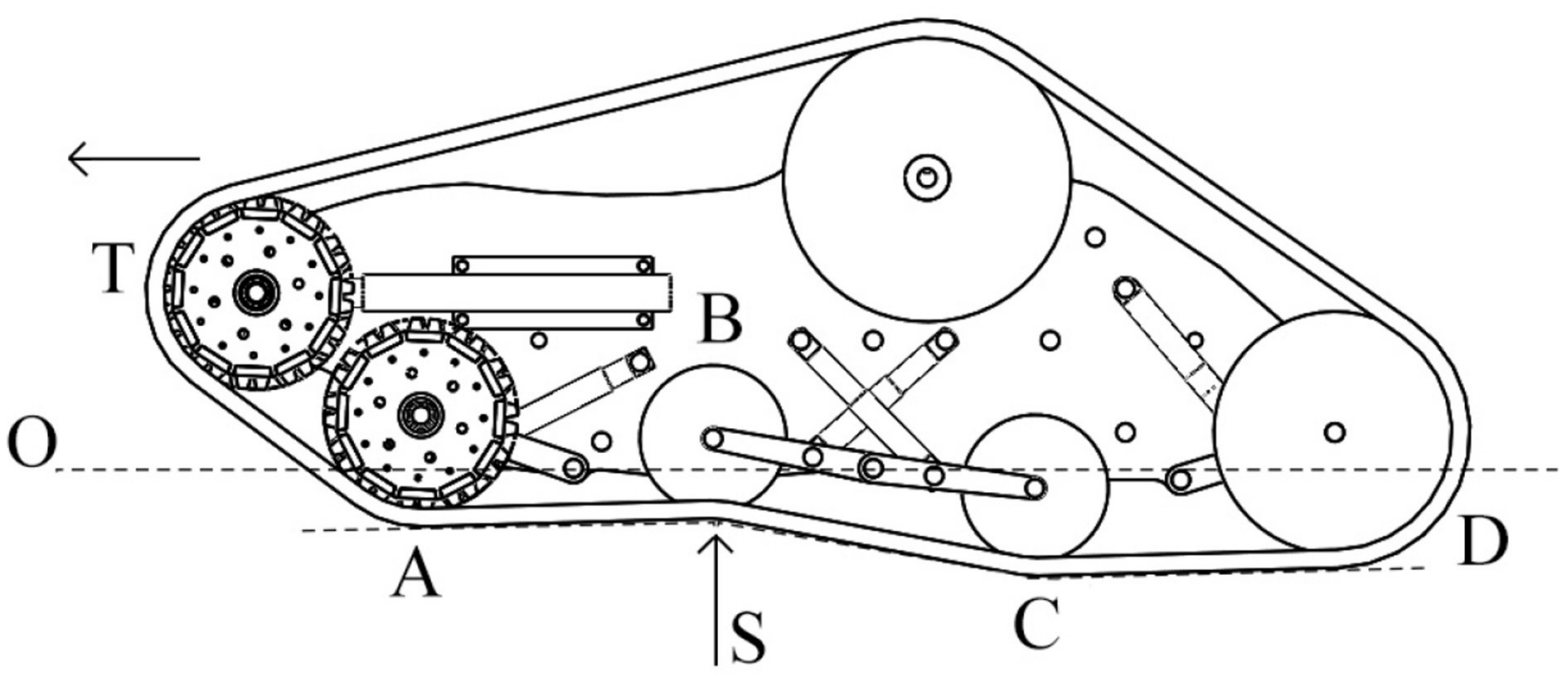
Example of suspension configuration.

**Figure 5:**
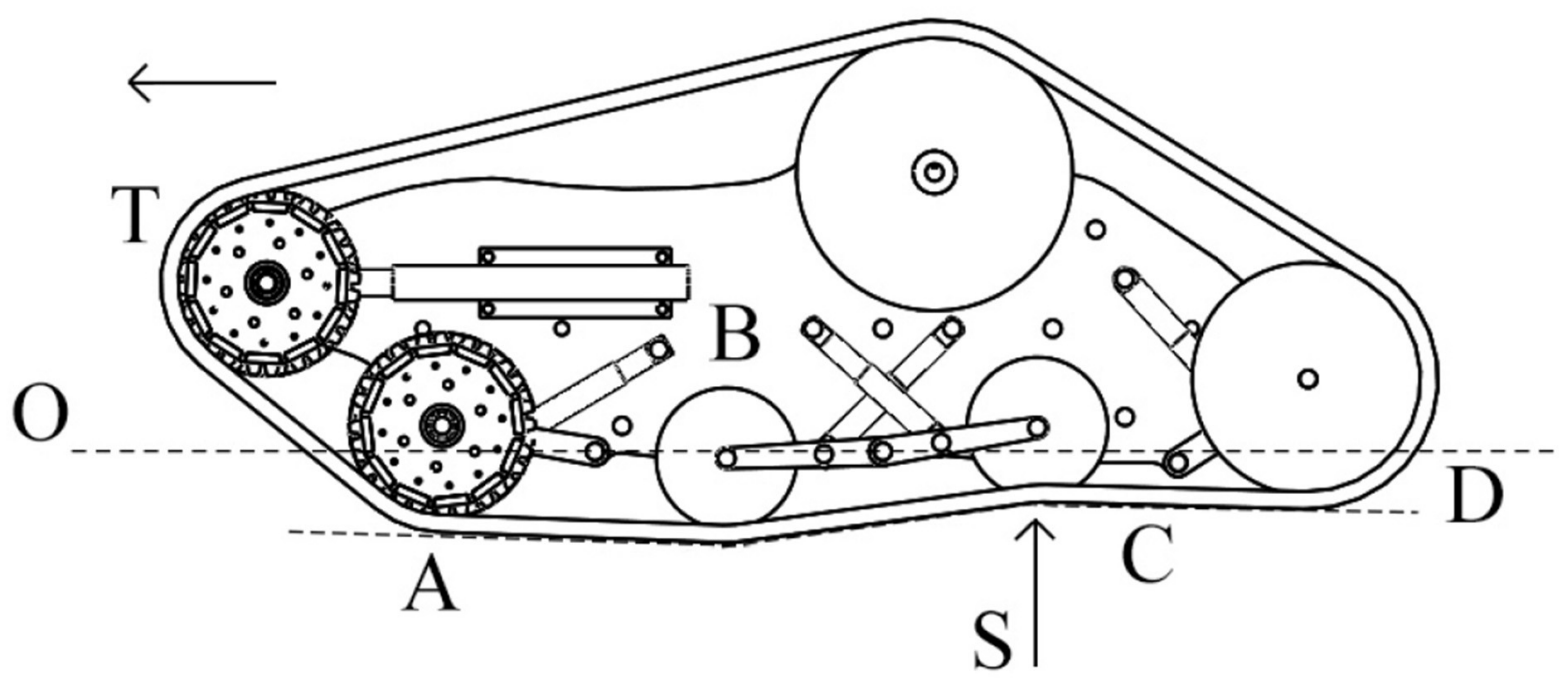
Another example of suspension configuration.

The passive suspension system of the vehicle performs multiple tasks such as maintaining the contact between the rubber tracks and terrain surface, providing the vehicle stability, and protecting the vehicle frame from all the shocks generated by the unevenness of the terrain. It works together with the rubber pad, the track idle wheels, the frame, and the suspension linkages to provide stability and somehow to physically separates the vehicle body from the rubber pad of the vehicle. Each track includes five idle wheels (A) and four single-arm suspension linkages (B), whose revolute joints (*O*^1^, *O*^2^, *O*^3^) are directly mounted on the robot frame, with four independent shock absorbers that allow one wheel to move upward and downward with a minimum effect on the other wheel as showed in Figure 2. The suspension system has been designed to provide sufficient vertical wheel motion so the vehicle can deal with roughness terrain. When an idle wheel contacts a bump, the suspension mechanism is able to allow sufficient vertical motion to avoid the wheel to continue to move upward, taking the frame with same high velocity and results on causing large vertical acceleration along the z-axis; this aspect is very important because it reduces the noise and the vibrations during the sensor acquisition. Figure 3 presents the case when the idle wheel moves in the vertical direction and get maximum values in bound (upward) where *H* = 0.10 m and rebound (downward) with *H* = − 0.05 m. A typical suspension configuration is represented in Figure 4 where it is possible to see what happens when the vehicle crosses a small bump **S**; in this case, as soon as the vehicle collides with the bump, the idle wheel **A** is forced to move up followed by the second idle wheel **B**. In order to keep the track belt tightened, the wheel **T** is pulled forward by the action of the spring tensioner while the wheel **C** is pulled down to maintain the belt in its position. Another typical suspension configuration is represented also in Figure 5 with the vehicle while passing a small bump **S**; in this situation, idle wheel A is very close to its normal position since it is moving on an horizontal plane while idle wheels **B** and **C** are diametrically opposed because they are trying to tighten the track belt under the action of their shock absorbers. The tensioner wheel appears to be moved outwards than in the previous configuration because the idle wheel **D** is moved up and decreases the track tension ahead since the vehicle is moving forward.

**Figure 6:**
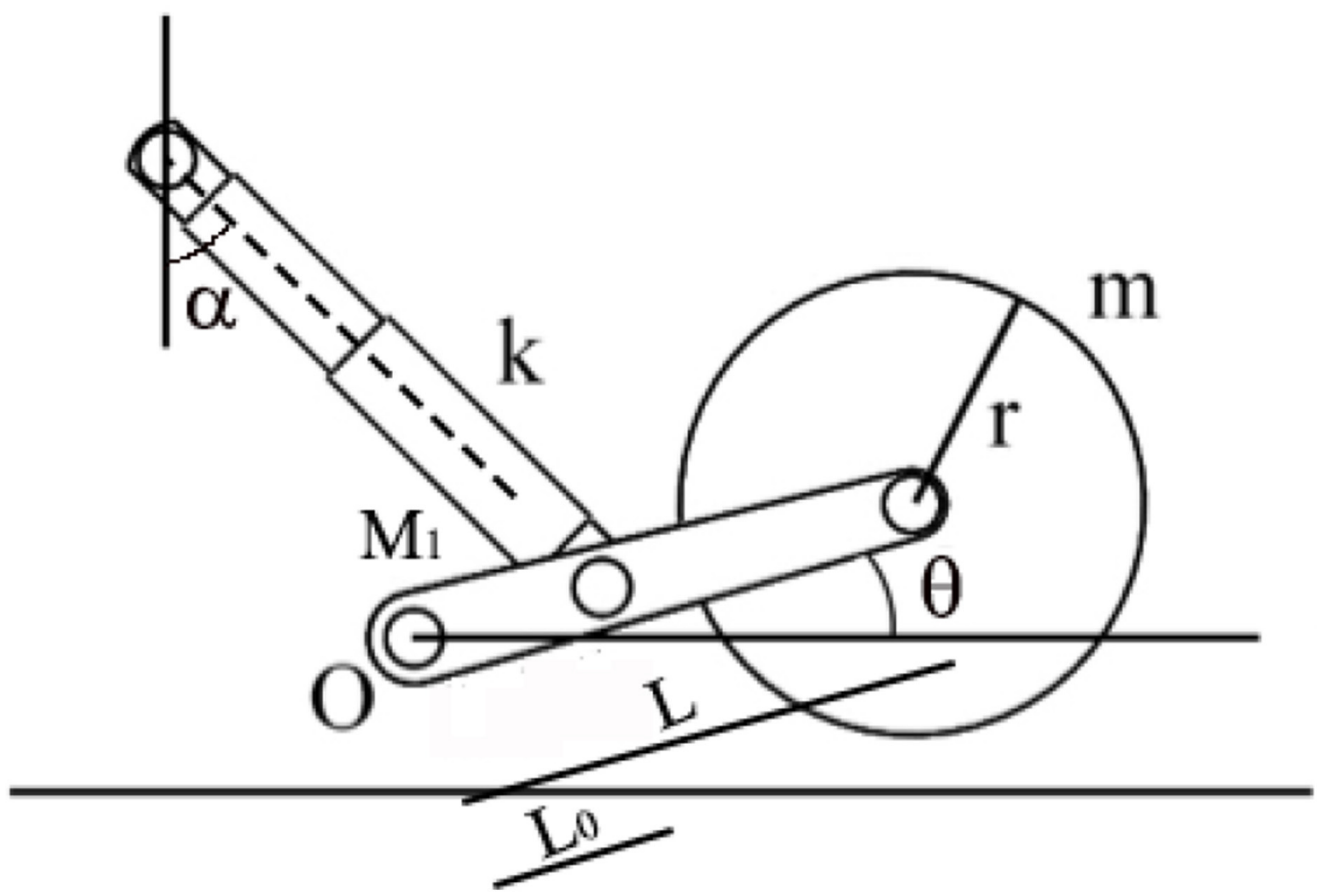
A simple subsystem made by a single-arm linkage with a shock absorber.

By considering a simplified suspension system as shown in Figure 6 where the presence of the sprung mass is neglected and the shock absorber has a spring constant of *k* = 37.27 N/mm, the linkage has mass *M*_1_ = 0.9 kg and length *L* = 0.1 m, and the idle wheel has a radius of *r* = 0.04 m, mass *m* = 0.5 kg, and stiffness *k*_*p*_, it is possible to write the equations to describe the subsystem behavior:


(1) $$\begin{array}{l} I\ddot{\theta }=-gLcos\theta \left(\frac{M_{1}}{2}+m\right)-k{\left(L_{0}cos\alpha \right)}^{2}sin\theta -L^{2}k_{p}sin\theta \end{array}$$

(2) $$\begin{array}{l} I=\frac{M_{1}}{3}L^{2}+mL^{2} \end{array}$$

Where I is the inertia expression for the assembly composed by the suspension linkage and the idle wheel, θ is the angle related to the angular displacement of the linkage, $\ddot{\theta }$ is its second derivative, and *O* is the pivot point for the rotational motion of the linkage. By considering the small oscillations, it is possible to rewrite the expression in (1) as follows:


(3) $$\begin{array}{l} I\ddot{\theta }=-gL\left(\frac{M_{1}}{2}+m\right)-k{\left(L_{0}cos\alpha \right)}^{2}\theta -L^{2}k_{p}\theta \end{array}$$

(4) $$\begin{array}{l} f_{n}=\frac{1}{2\pi }\sqrt{\frac{6\left(k{\left(L_{0}cos\alpha \right)}^{2}+k_{p}L^{2}\right)}{2M_{1}L^{2}+6mL^{2}}} \end{array}$$

The last equation in (4) is used to express the natural frequency associated with the suspension system.

### 2.3. The Software Architecture

**Figure 7:**
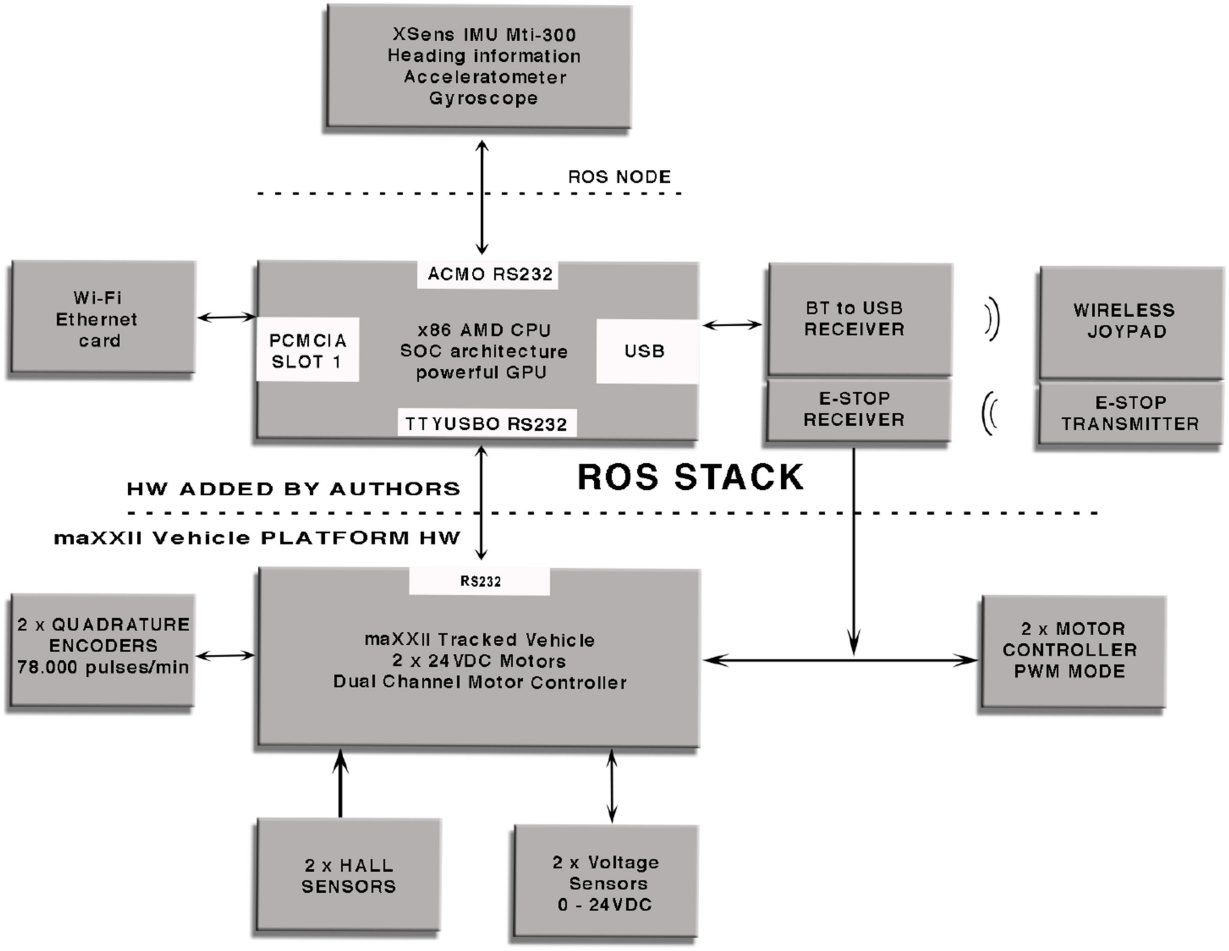
Functional block diagram for the used vehicle.

ROS (Robot Operating System) ROS (2007) is used both to control the vehicle and to read data from all the sensors since it allows the user to easily employ a large set of libraries, filters and tools to acquire and process the data coming from the sensors; moreover, the user can send Twist commands to the vehicle and makes it move depending on the linear components for the (x, y, z) velocities and on the angular components for the angular rate for the (x, y, z) axes. The system runs on an x86 AMD CPU based on SOC architecture and integrates a powerful GPU for graphical processing and a Wi-Fi card for remote connection; the operating system used for the experimental tests was Ubuntu with a ROS server in order to exchange messages with a remote machine used as a client. Figure 7 provides a functional block diagram that shows the hardware layer used for this research work which includes an inertial sensor, Mti-300 by XSens, a laser sensors, the LMS-111 by SICK, two optical encoders, two hall sensors, two voltage sensors and the RTK GPS), the Wi-Fi module needed for the remote communication with the vehicle, the Bluetooth receiver which enables the vehicle to be manually controlled and the dual channel motor controller. A specific ROS node has been designed in C++ in order to let the vehicle to communicate with the sensors while another one has been developed to send the locomotion instructions to the motor controller and to send the acquired values from sensors over the Wi-Fi network.

## 3. Vehicle Terrain Interaction

### 3.1. The Kinematic Model of the Vehicle

**Figure 8:**
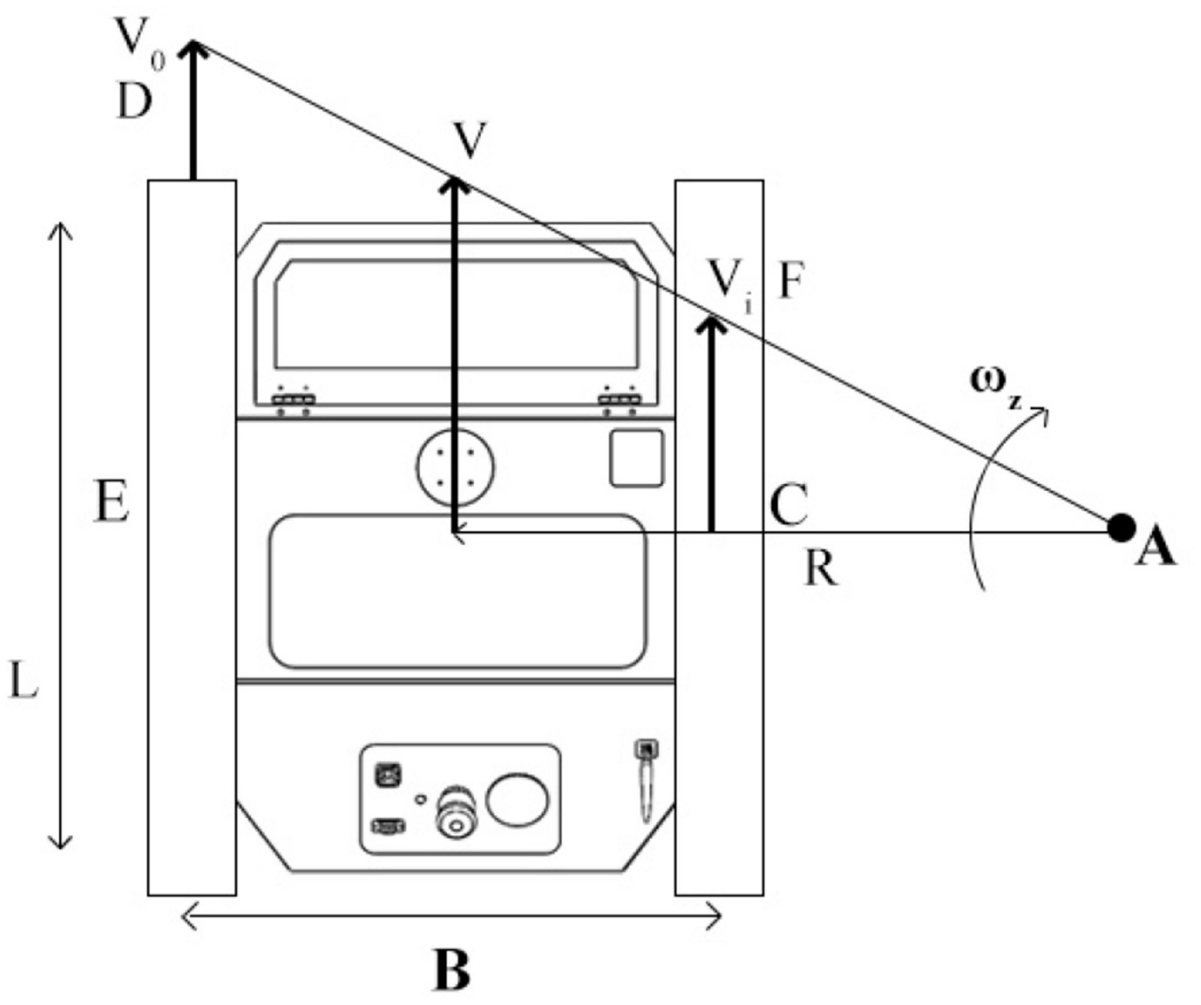
The kinematic for a skid-steering tracked vehicle during a clockwise steering maneuver where B is the width of the vehicle body, *V*0 is the velocity for the outer track and Vi is the velocity for the inner track.

Driving systems based on the skid-steering method are usually used on tracked vehicles such as caterpillars and military tanks for off-road applications. For this kind of vehicles, the left and right tracks can move at different velocity both in forward and reverse mode depending on the sprocket angular velocity and direction. Due to the complex track pads and terrain interactions, it is very hard to accurately describe a correct kinematic model for skid-steer mobile vehicles. In this case, a correct study about the wheel slip has a key role in kinematic and dynamic modeling of skid-steer mobile vehicles; this is because the slip information can describe a relation between the wheel angular velocity and the linear motion of the vehicle platform. Skid-steer vehicle localization applications, like dead reckoning, strictly rely on the determination of the slippage information even if this information can be also used to extract and investigate terrain conditions. Figure 8 shows the kinematics principles of a skid-steer vehicle while a clockwise steering by considering a right-hand vehicle fixed coordinate system with its origin placed in the vehicle center of mass. By using the similar triangle properties, the equation to measure the steering radius can be obtained by considering the proportion between each side of the two triangles *AFC* and *ADE* as in Equations (5) and (6).


(5) $$\frac{v_{0}}{vi}=\frac{R+\frac{B}{2}}{R-\frac{B}{2}};R=\frac{\frac{B}{2}(\frac{V_{0}}{V_{i}}+1}{\left(\frac{v_{0}}{v_{i}}-1\right)}=\frac{B}{2}(\frac{v_{0}+v_{i}}{v_{0}-v_{i}})$$

(6) $$\begin{array}{l} \omega_{z}=\frac{v_{o}+v_{i}}{2R}=\frac{v_{i}\left(\frac{V_{o}}{V-i}-1\right)}{B} \end{array}$$

It should be noted that the steering radius calculated in Equation (5) is considered under the assumption that no slippage effects are generated between the idle wheels and the rubber track and between the rubber pads and the ground during the turning maneuver. However, in the real world, skidding and slipping effects between idle wheels, rubber tracks, and ground surfaces can be observed for all skid-steering vehicles since slipping is necessary when a vehicle heading change is needed. As a consequence, even at low steering angular velocity, a traditional kinematics approach is not enough to correctly describe the vehicle position in the environment. The difference between the vehicle forward velocity and the sprocket angular velocity can be obtained as result of the longitudinal slip effect *i*, which can be well described by:


(7) $$\begin{array}{l} i=\left(1-\frac{V}{r\omega }\right)100; \end{array}$$

(8) $$\begin{array}{l} R^{\prime }=\frac{B}{2}\left(\frac{v_{0}\left(1-i_{0}\right)+v_{i}\left(1-i_{i}\right)}{v_{0}\left(1-i_{o}\right)-v_{i}\left(1-i_{i}\right)}\right) \end{array}$$

while the new yaw rate estimate will be as showed in the following Equation (9):


(9) $$\begin{array}{l} \omega_{z}^{\prime }=\frac{vi\left(\frac{v_{o}\left(1-i_{0}\right)}{v_{i}}-\left(1-i_{i}\right)\right)}{B} \end{array}$$

### 3.2. The Extended Kalman Filter for the Equivalent Track

Despite the fact that several research studies rely on skidding estimation for vehicle localization (Martinez et al., 2005) and path planning (Pentzer et al., 2014), an accurate relation between the longitudinal skidding effect and the vehicle behavior has not been acquired yet. Surely, it is possible to consider almost all the skidding and slipping effects as a result of the interaction between both left and right rubber tracks and the ground surface; it is even worth noting that the slipping effects introduce an error to the encoder readings that cannot be used to calculate the vehicle position. In this work, the concept of equivalent track, previously introduced by the authors (Reina and Galati, 2016), is used as a parameter to describe the terrain conditions and to classify it depending on the amount of slippage introduced into the vehicle system. By expressing the angular velocity of the vehicle ω_*z*_ as:


(10) $$\begin{array}{l} \omega_{z}=\Delta V\frac{1}{B_{s}} \end{array}$$

where *B*_*s*_ is the estimated equivalent track, it is possible to implement a state observer using an EKF by extending Equation (10) to a discrete-time state-space model (Reina et al., 2017b) where the parameters values change like in a random walk:


(11) $$\begin{array}{l} x_{k+1}=x_{k}+\omega_{k};z_{k+1}=H_{k+1}x_{k+1}+v_{k+1}; \end{array}$$

where *x*_*k*_ = 1/*B*_*s*_ is the state variable at time **k**, while *z*_*k*+1_ is the observation, i.e., ω, and *H*_*k*+1_ the measurement coefficient, i.e., Δ*V*, at time **(k+1)**. The EKF estimation operates through the prediction-correction cycle expressed by:

Prediction:


(12) $$\begin{array}{l} {\hat{x}}_{k+1}^{-}=A_{d}{\hat{x}}_{k}+B_{d}u_{k} \end{array}$$

(13) $$\begin{array}{l} P_{k+1}^{-}=H_{k+1}xk+1+v_{k+1} \end{array}$$

Correction:


(14) $$\begin{array}{l} K_{k+1}=P_{k+1}^{-}H_{d}^{T}{\left(H_{d}P_{k+1}^{-}H_{d}^{T}+R\right)}^{-}1 \end{array}$$

(15) $$\begin{array}{l} {\hat{x}}_{k+1}={\hat{x}}_{k+1}^{-}+K_{k+1}\left(z_{k+1}-H_{d}{\hat{x}}_{k+1}^{-}\right) \end{array}$$

(16) $$\begin{array}{l} P_{k+1}=\left(I-K_{k+1}H_{d}\right)P_{k+1}^{-} \end{array}$$

where ${\hat{x}}_{k+1}^{-}$ is the predicted state vector, $P_{k+1}^{-}$ is the variance matrix for ${\hat{x}}_{k+1}^{-}$, *K*_*k*+1_ is the gain matrix, ${\hat{x}}_{k+1}$ is the updated state vector, and *P*_*k*+1_ is the updated error covariance estimate. The slip track estimation is calculated only during turning maneuvers since the filter is switched off during straight motion due to the lack of excitation. It should be noted that the slip track measurement remains bounded. When the vehicle executes straight driving, both numerator and denominator in equation $B_{s}=\Delta V\omega_{z}^{-1}$ are infinitesimals of the same order and this results in finite values of *B*_*s*_.

### 3.3. Motor Currents Analysis

Unlike it happens in wheeled vehicles where the portion of the tire's tread that touches the terrain surface is very small while the ground pressure can reach very high values, the tracked vehicles are characterized by a larger fingerprint on the soil surface and by a lower ground pressure. Moreover, the value of the maximum tractive effort *F*_*max*_ that can be generated by a tracked vehicle is produced by the shear stress of the terrain, τ_*max*_, and the contact area A as showed in the following equation:


(17) $$\begin{array}{l} F_{max}=A\tau_{max}=Ac+Wtan\phi \end{array}$$

where *A* = 0.220*m*^2^ is the contact area for both tracks and *W* = 392*N* is the normal load while *c* and ϕ are strictly related to the terrain type. Since, in electrical vehicles, the tractive effort, the thrust, and the torque can be considered as roughly proportional to the DC motor current:


(18) $$\begin{array}{l} T_{r}=\tau k_{t}I \end{array}$$

where *T*_*r*_ is the motor torque constant and τ = 60 is the gearbox ratio. So, by measuring the left and the right motor currents during straight motion at constant velocity, it is possible to have an indirect estimation of the motion resistance for a specific terrain condition, given the track geometry and the vertical load. Due to the particular track design, it is worth noting that the amplitude of current peaks and their period change according with the physical characteristics of each terrain. The irregularities of the terrain generate a different power transfer to the tracks by requesting more or less motor torque. On asphalt, the motor current values present regular peaks and period since the surface is almost flat and does not include roughness; in this case, the electrical current amplitude is limited. On gravel or rocks soils, the current values present several high peaks due to the presence of debris and irregularities while sand terrain is characterized by low peaks, but by the highest current amplitude. This happens because sand has higher deformability than the asphalt and offers a larger contact area for the tracks.

### 3.4. Vertical Accelerations

The vibration response of tracked vehicle on the terrain is quite different from the response of the vehicle on wheels. So, in order to define the accurate dynamic model of a tracked vehicle is very important to study the vehicle vibration response. In linear systems, a direct linear relationship between input and output signals exists. Typically, a vehicle system which is defined by its transfer function, takes in account the input representing the terrain irregularities and generates an output representing the vibration of the vehicle. In this case, the frequency response function can be defined as the ratio of the output to input under steady-state conditions. If it is possible to consider a simplified single-degree-of-freedom model for the vehicle and both the input and output values can be expressed in terms of displacements and the vibration of the sprung mass as output is calculated in term of accelerations, then the modulus of the transfer function *H*(*f*) is expressed as follows:


(19) $$\left|\left(H(f\right)\right|={\left(2\pi f\right)}^{2}\sqrt{\frac{1}{1-{\left(\frac{f}{f_{n}}\right)}^{2}}}$$

where *f* is the frequency of excitation and *f*_*n*_ is the natural frequency of the system. The damping ratio is not included in Equation (19) since the shock absorber used by the vehicle works only with a spring without dampers. When the transfer function of a specific system is known, then, it is possible to express the relation between the power spectral density of the input *S*_*g*_(*f*) and the power spectral density of the output *S*_*v*_(*f*) of the whole system as follows:


(20) $$\begin{array}{l} S_{v}\left(f\right)=|H{\left(f\right)}^{2}|S_{g}\left(f\right) \end{array}$$

When considering linear systems, this relation shows how the output power spectral density is associated to the input power spectral density through the square of the modulus of the transfer function. The power spectral density defines how the power of a signal is distributed over frequency and it is strictly correlated to the interaction between the terrain profile and the track belt and between the belt and the track sprocket. In contrast to the wheeled vehicles which usually present only a single peak in their frequency answer, the vehicle used for this study showed four distinct and separate peaks and four odd harmonics in total. The study of this important aspect allows finding the proper fingerprint for each terrain profile.

The power spectral density function has been used also to study the motor current behavior during steady-state conditions when the vehicle moves straight for at least 30 s and at its maximum speed over different terrains. This gives an overall idea about the spectral energy distribution and the current signature for each terrain profile.

## 4. Experimental Results

### 4.1. Field Experiments

**Figure 9:**
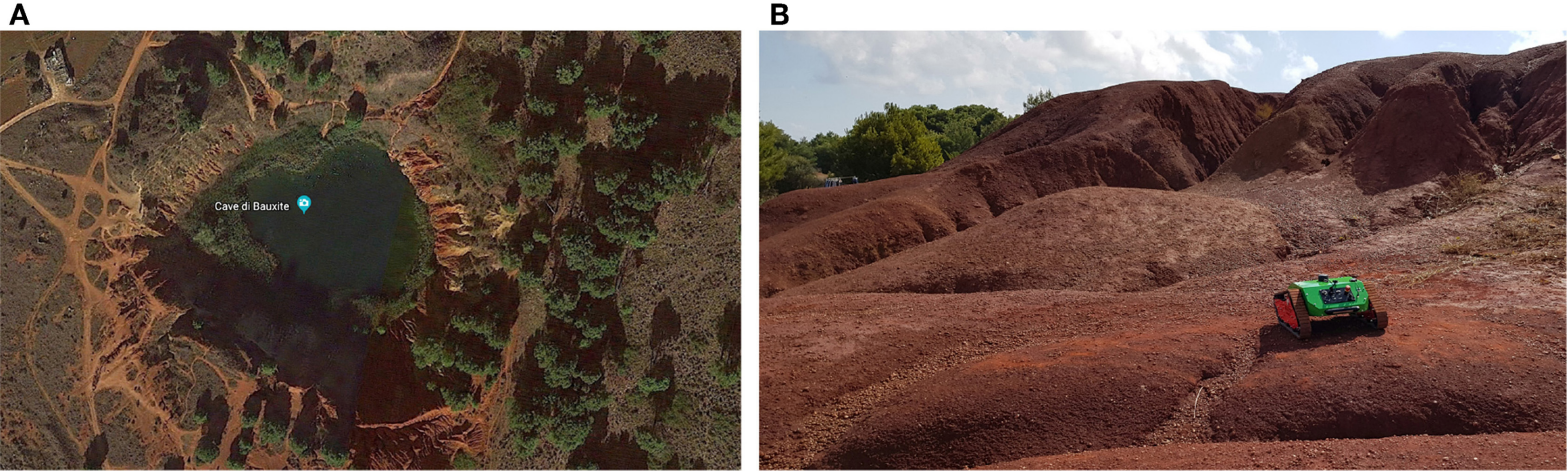
**(A)** Aerial view of the test field in a Bauxite pit, **(B)** the vehicle moving on natural bumps.

In order to validate the method and acquire the data, several tests were performed on different terrain surfaces: sand, gravel, mud and asphalt. Figure 9 shows an aerial view of the test field taken from Google Earth (40° 7′ 56.0856″ N, 18° 30′ 2.2356″ E) used for the experimental campaigns (on the left) which is located in Otranto, Italy and a view of the vehicle moving on small natural bumps (on the right). In all the experiments, the vehicle “maXXII” was forced to perform two main motion primitives and, in particular, a straight line at constant speed of 0.75 m/s followed by a steering maneuver at a constant rate of turn of 45 deg/s. During each test, a set of data was recorded by using the rosbag utility provided by ROS by including the motor currents, the angular velocities for the track sprockets and the accelerations along the vertical axis at a frequency of *F*_*s*_ = 120 Hz. For each terrain, a “fingerprint” has been defined by combining specific values of electrical currents, equivalent track and power spectral density of both electrical currents and vertical accelerations; after this, a classification learner tool was used to train a model over the classified data.

### 4.2. Terrain Estimation

**Figure 10:**
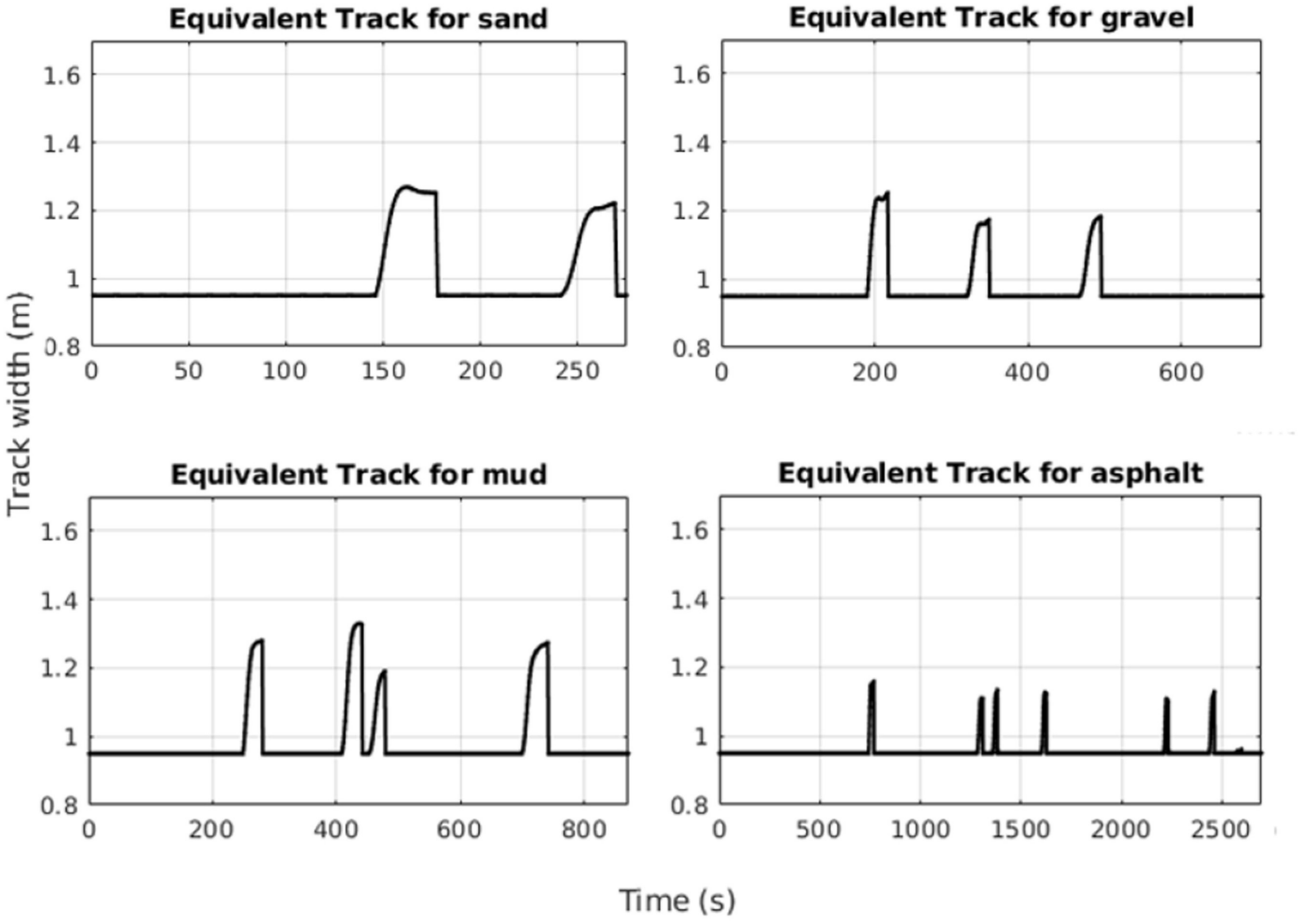
Equivalent track values for sand, gravel, mud, and asphalt.

The registered values for the equivalent track are showed in Figure 10 where it is possible to check the vehicle behavior during turning operations over sand, gravel, mud and asphalt. All the main values are reported in Table 1 and the maximum equivalent track of 1.329 m has been reported during the test over the mud where the wet surface generates the highest slipping effect while asphalt reported the lowest average value with only 1.159 m.

**Table 1:** Equivalent track values for different terrains.

**Equivalent track values [m]**			
**Sand**	**Gravel**	**Mud**	**Asphalt**
1.269	1.252	1.329	1.159

**Figure 11:**
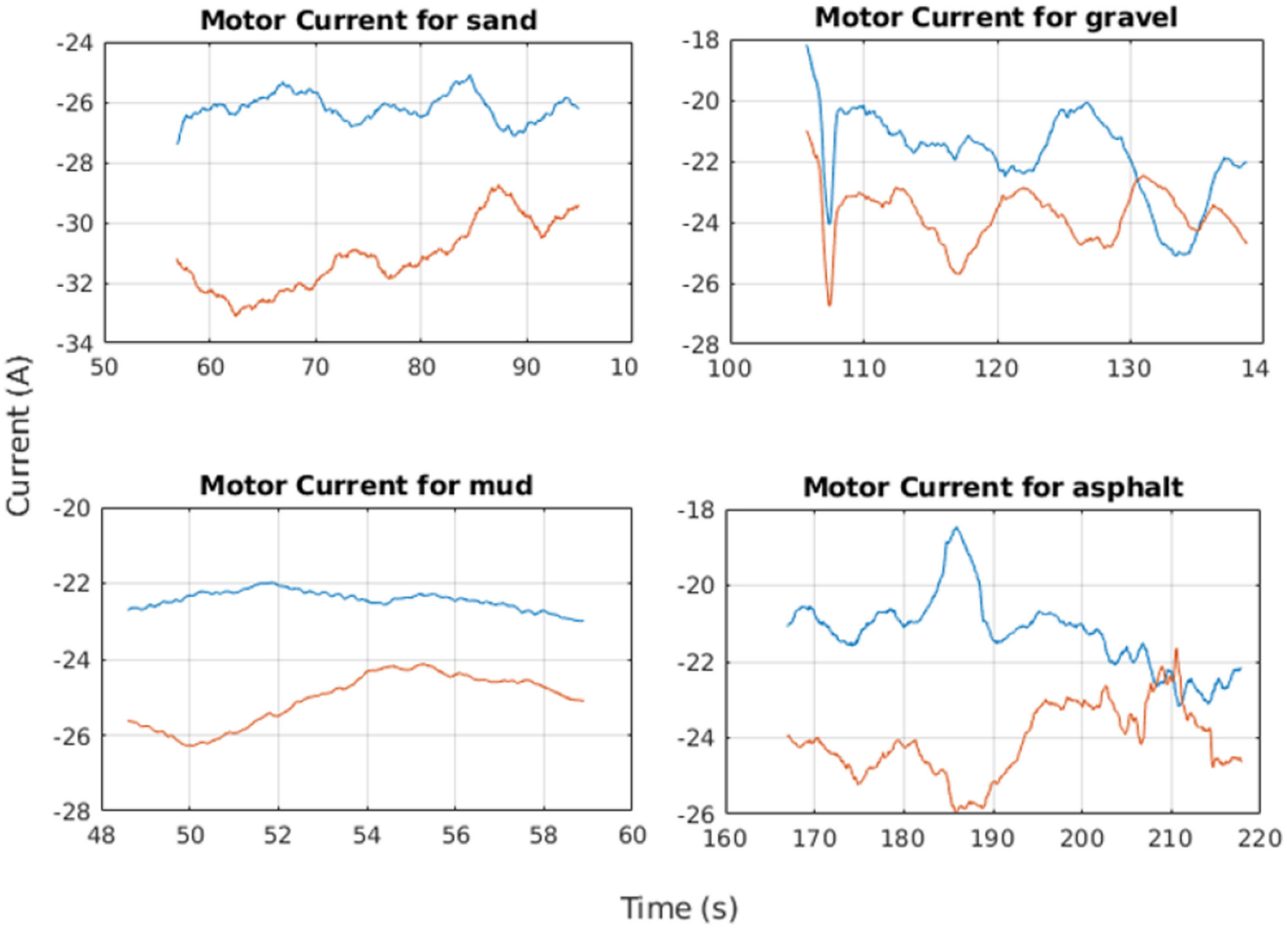
Motor current values for sand, gravel, mud, and asphalt.

Moreover, the motor currents presented a different behavior on each terrain by showing wide and narrow current peaks over the asphalt and gravel and almost a flat trend on mud and sand. In these two last cases, the current amplitude was higher than on asphalt and gravel since sand and mud terrain are more flexible and offer a larger contact area for the tracks that cause a request for a higher tractive effort as showed in Figure 11 where the blue line is referred to the left motor current while the red line is referred to the right motor current; the offset between both currents is due to different intrinsic motor characteristics and to power dissipation. Average values for current are reported in Table 2 and are related to straight paths.

**Table 2:** Equivalent track values for different terrains.

**Motor currents [A]**			
**Sand**	**Gravel**	**Mud**	**Asphalt**
26.17	21.77	24.01	21.23

**Figure 12:**
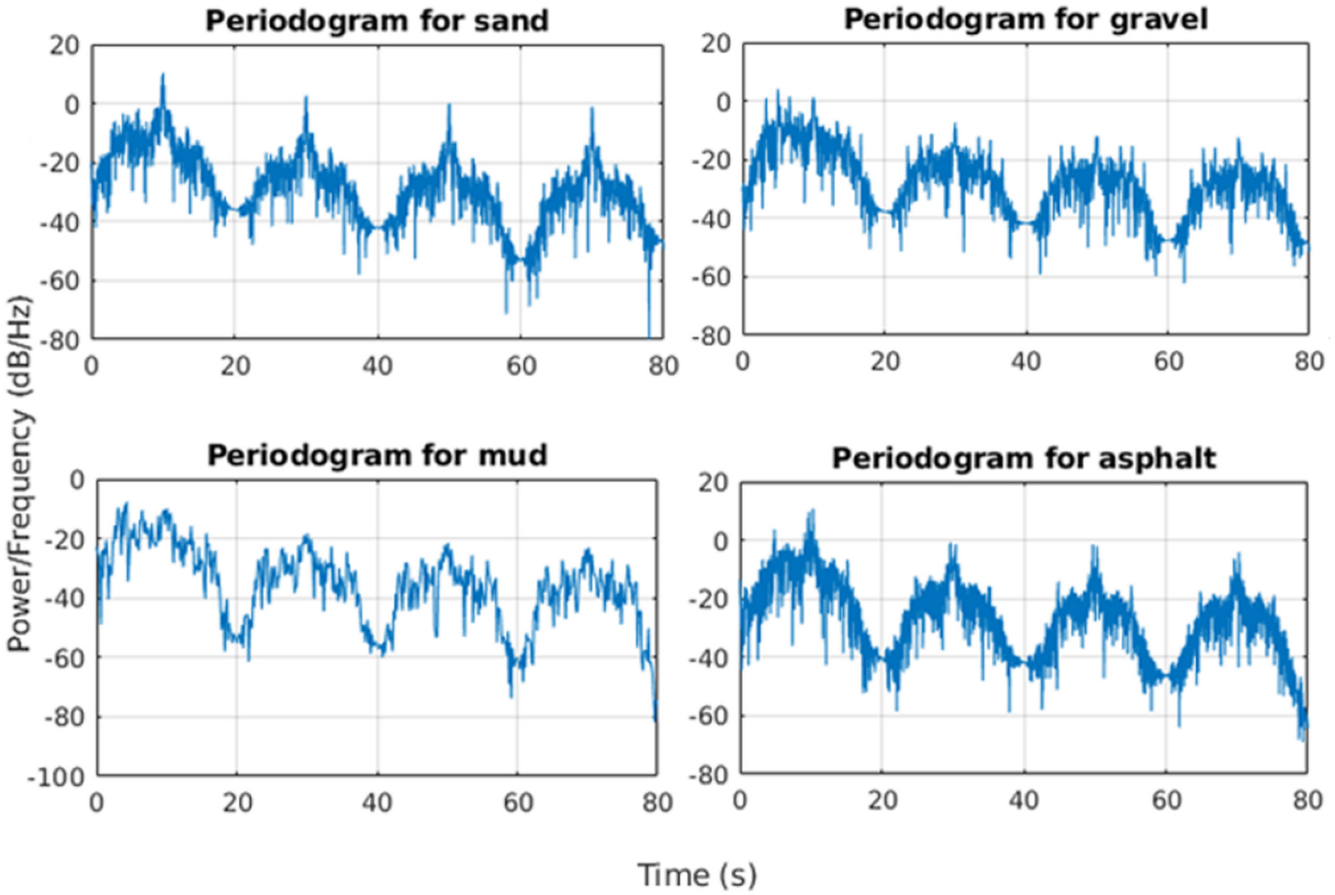
Power spectral density values for sand, gravel, mud, and asphalt.

The power spectral density was computed over sample with a time length of *t* = 10 s by using the accelerations along the Z-axis of the vehicle recorded from the inertial sensor and shows how the tracks interact with the terrain profile as it is possible to see in Figure 12.

In particular, it is worth noting that the higher power spectral density value was registered at around 10 Hz for all the tests by showing a sort of vehicle intrinsic periodicity as showed in Table 3. The specific design of the track generates in total four harmonics for each terrain: the first one is centered at 10 Hz for all the terrains except for the mud. The third harmonic is located at around 30 Hz, the fifth at 50 Hz and the seventh harmonic at 70 Hz. Mushy mud decreases the mobility of the whole track and this induces a very specific slower frequency response while asphalt provides a much more responsive and reactive terrain profile.

**Table 3:** Typical values for the PSD of body vertical acceleration.

**PSD for motor currents [dB]**			
**Sand**	**Gravel**	**Mud**	**Asphalt**
−14.23	−15.15	−13.22	−15.8

The previous method based on the power spectral density function has been applied also on motor currents during straight motion in order to add and enhance information about the profile signature for each terrain.

For this specific situation, the power spectral density has been computed by using the Welch's method which relies on the concept of using periodogram spectrum estimates as the result of converting a signal from the time domain to the frequency domain to reduce the noise in the estimated power spectra in exchange for reducing the frequency resolution. The method is applied on both the left and right motors; the average of their PSD amplitudes has been considered as an additional parameter for the terrain characterization. Table 4 reports some typical values for the PSD amplitudes applied on motor currents over different terrains.

**Table 4:** Power spectral density.

	**Sand**	**Gravel**	**Mud**	**Asphalt**
PSD [dB/Hz]	10.37	1.797	−6.794	10.67
1^{st}HM [Hz]	10.6	9.997	7.316	10.29
PSD [dB/Hz]	2.637	−7.605	−18.29	−0.778
3^{st}HM [Hz]	30.06	29.99	29.89	29.74
PSD [db/Hz]	−0.108	−11.63	−21.78	−1.494
5^{st} HM [Hz]	50.05	49.98	49.87	49.72
PSD [dB/Hz]	−1.179	−13.68	−23.61	−4.079
7^{st} HM [Hz]	70.04	69.98	69.85	70.29

### 4.3. The Classification Algorithm

**Figure 13:**
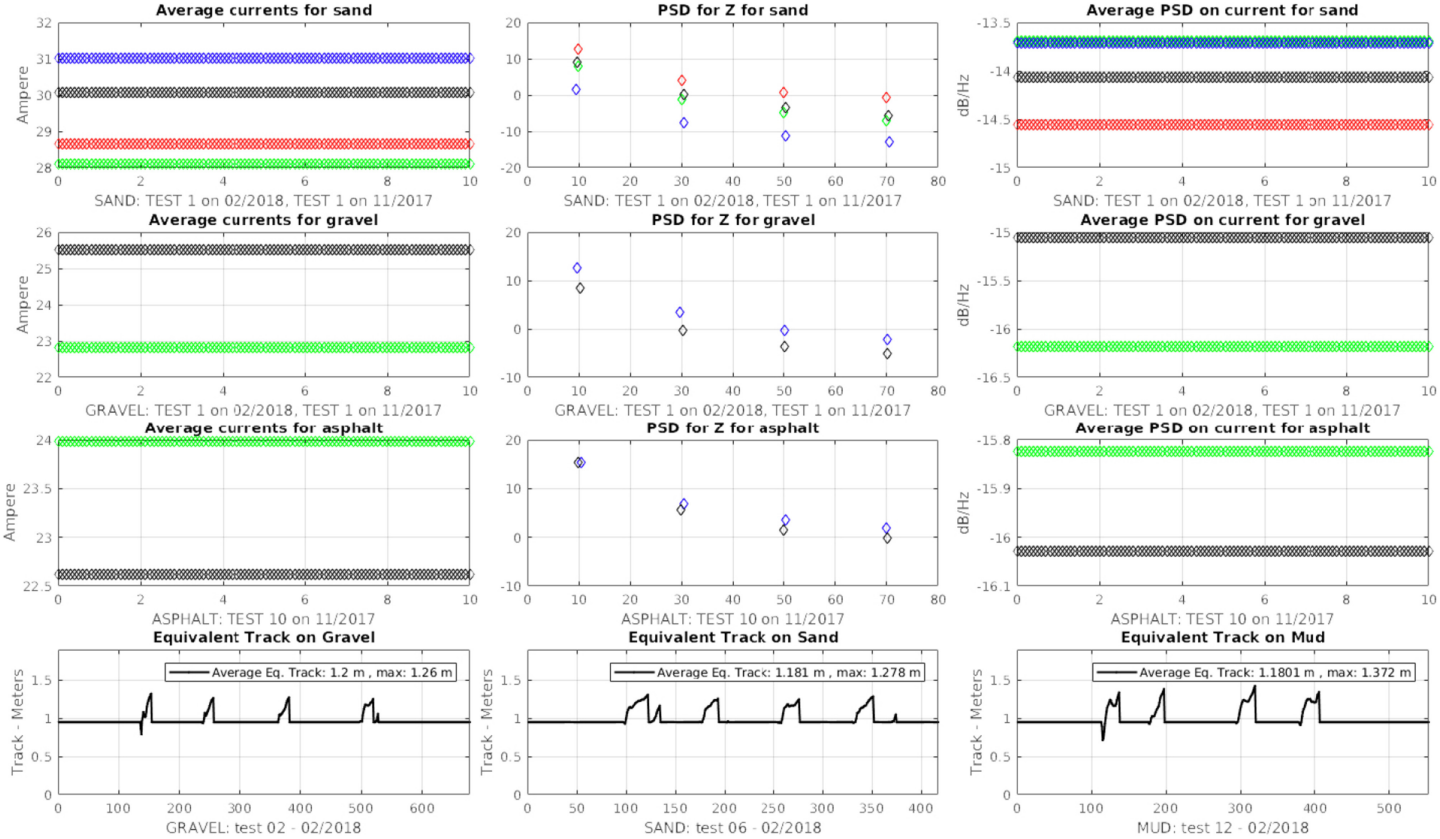
Reference values for some terrain profiles.

After 100 real world testing campaigns, the average values of motors current and power spectral density both for accelerations along the Z-axis and for motors current have been recorded for each terrain profile by running the tests for *t* = 40 s over a straight line on a specific terrain while the equivalent track has been acquired during a steering maneuver on the same terrain, with *t* = 10 s, in order to generate a relational database where each terrain is formally described by a range of average values. Figure 13 shows samples for the average values related to some of the main terrain profiles. Data has been stored in a text file where each sample array was composed by four numerical values (equivalent track, motors current, PSD over accelerations, PSD over currents). Subsequently, a probabilistic graphical model based on Bayesian network representing a group of variables and their conditional dependencies by using a Directed Acyclic Graph (DAG) has been designed along a ROS node to compare the online acquired sensor data against the dataset stored into the database. This ROS node has been used to output on-line estimations about the terrain profile by running the probabilistic algorithm on the real-time data coming from the vehicle sensors.

## 5. Conclusion

**Figure 14:**
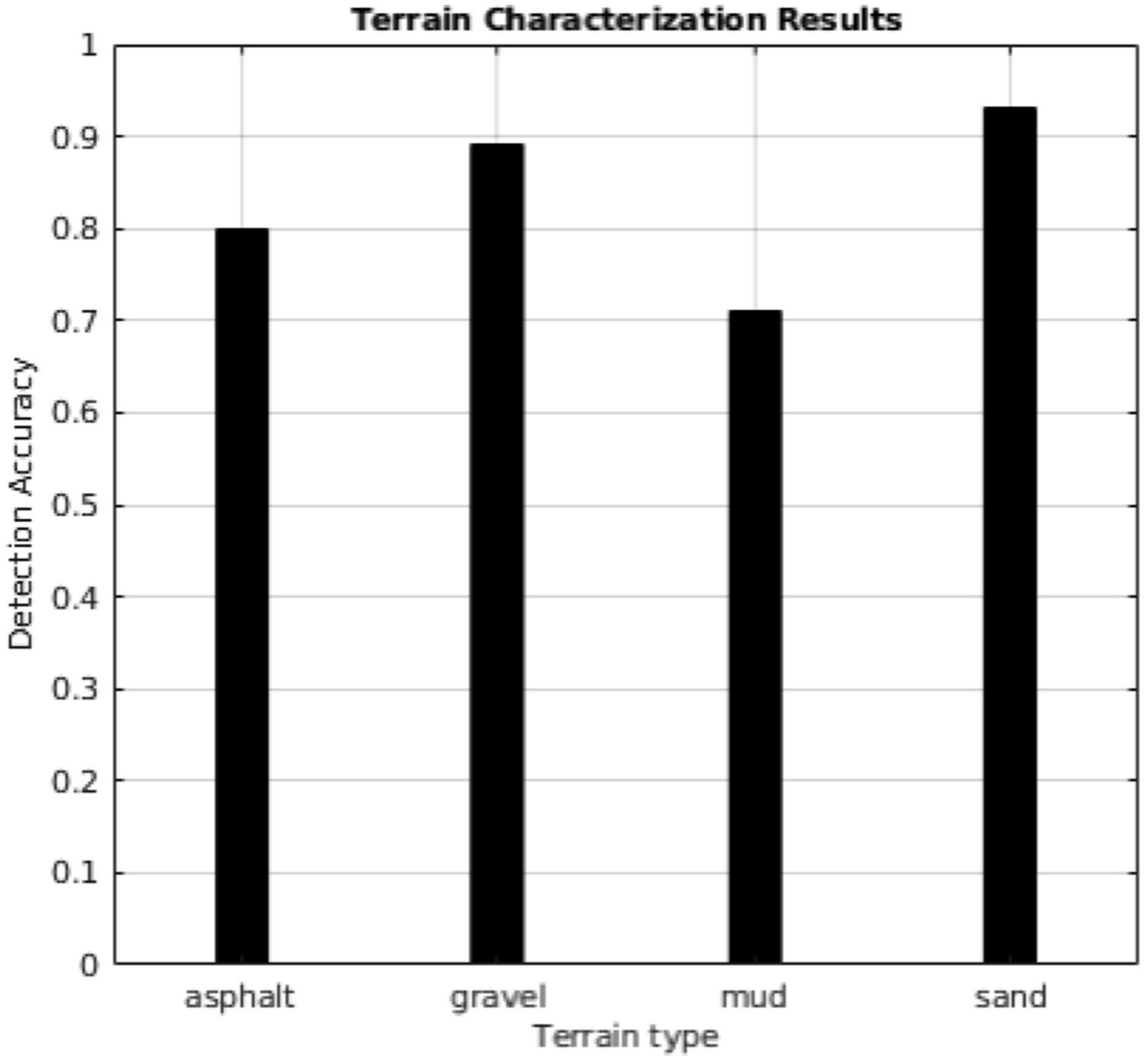
The accuracy of the proposed classification method.

In this paper, a method for terrain characterization was presented that used a tracked skid-steer vehicle with independent passive suspensions. It is based on the estimation of three parameters that depend on the terrain: motor currents, equivalent track and power spectral density. These parameters can be measured during normal driving by monitoring the vehicle motion, the motor currents and the accelerations along the vertical axis. A model-based Kalman observer was introduced to estimate the slip while the power spectral density function was used to study the vehicle vibration response for different terrain profiles. Experiments demonstrate that the classifier can effectively distinguish four types of terrain profiles including asphalt, gravel, mud, and sand with a high accuracy of over 89% for gravel and sand as reported in Figure 14. Mud detection presents a rate of success of about 72% and needs further investigations due to its unpredictable nature mainly on tracked vehicles. A further follow up to this study will consider also the suspension displacement and position by reading data from linear potentiometers mounted in parallel on each shock absorber in order to investigate the frequency response for each suspension.

## Data Availability

The datasets generated for this study are available on request to the corresponding author.

## Author Contributions

Both authors made significant contributions to the conception and design of the research. They equally dealt with data analysis and interpretation, and writing of the manuscript.

### Conflict of Interest Statement

The authors declare that the research was conducted in the absence of any commercial or financial relationships that could be construed as a potential conflict of interest.
