# Corrigendum: Terrain awareness using a tracked skid-steering vehicle with passive independent suspensions

**DOI:** 10.3389/frobt.2023.1204228

**Published:** 2023-05-30

**Authors:** Rocco Galati, Giulio Reina

**Affiliations:** Department of Engineering for Innovation, University of Salento, Lecce, Italy

**Keywords:** mobile robotics, terrain recognition, tracked vehicles, skid-steering, articulated suspension system

In the published article, there was an error in section **2. Materials and Methods**, *2.2 The Suspension System*, paragraph two, regarding a term in Eq. 2 that was used to describe the simplified suspension system, as represented in Figure 6. This term was erroneously added to the equation because of an oversight, and in any case, it has not been used to elaborate the experimental data.

The paragraph previously stated:

“By considering a simplified suspension system as showed in Figure 6 where the presence of the sprung mass is neglected and the shock absorber has a spring constant of k = 37.27 N/mm, the linkage has mass *M*_1_ = 0.9 kg and length L = 0.1 m, the idle wheel has a radius of r = 0.04 m, mass m = 0.5 kg and stiffness *k*_*p*_, it is possible to write the equations to describe the subsystem behavior:

$$\;I\Theta^{\prime \prime }=-\frac{1}{2}gcos\theta \left(M_{1}L+mg\left(L+r\right)\right)-kL_{0}^{2}cos\theta sin\theta$$ (1)

$$I=\frac{M_{1}}{3}L^{2}+\frac{m}{2}r^{2}+mL^{2}$$ (2)

Where I is the inertia expression for the assembly composed by the suspension linkage and the idle wheel, *θ* is the angle related to the angular displacement of the linkage, $\ddot{\theta }$ its second derivative, and *O* is the pivot point for the rotational motion of the linkage. By considering the small oscillations, it is possible to rewrite the expression in (Eq. 1) as:

$$I\ddot{\theta }=-gL\left(\frac{M_{1}}{2}+m\right)-k{\left(L_{0}cos\alpha \right)}^{2}\theta -L^{2}k_{p}\theta$$ (3)

$$f_{n}=\frac{1}{2\pi }\sqrt{\frac{6\left(k{\left(L_{0}cos\alpha \right)}^{2}+k_{p}L^{2}\right)}{2M_{1}L^{2}+3mr^{2}+6mL^{2}}}$$ (4)

The last equation in (4) is used to express the natural frequency associated with the suspension system.”

The corrected paragraph appears as follows:

“By considering a simplified suspension system as shown in Figure 6, where the presence of the sprung mass is neglected and the shock absorber has a spring constant of k = 37.27 N/mm, the linkage has mass *M*_1_ = 0.9 kg and length L = 0.1 m, and the idle wheel has a radius of r = 0.04 m, mass m = 0.5 kg, and stiffness *k*_*p*_, it is possible to write the equations to describe the subsystem behavior:

$$I\ddot{\theta }=-gLcos\theta \left(\frac{M_{1}}{2}+m\right)-k{\left(L_{0}cos\alpha \right)}^{2}sin\theta -L^{2}k_{p}sin\theta ,$$ (1)

$$I=\frac{M_{1}}{3}L^{2}+mL^{2},$$ (2)

where I is the inertia expression for the assembly composed by the suspension linkage and the idle wheel, *θ* is the angle related to the angular displacement of the linkage, $\ddot{\theta }$ is its second derivative, and *O* is the pivot point for the rotational motion of the linkage. By considering the small oscillations, it is possible to rewrite the expression in (Eq. 1) as follows:

$$I\ddot{\theta }=-gL\left(\frac{M_{1}}{2}+m\right)-k{\left(L_{0}cos\alpha \right)}^{2}\theta -L^{2}k_{p}\theta ,$$ (3)

$$f_{n}=\frac{1}{2\pi }\sqrt{\frac{6\left(k{\left(L_{0}cos\alpha \right)}^{2}+k_{p}L^{2}\right)}{2M_{1}L^{2}+6mL^{2}}}.$$ (4)

The last equation in (4) is used to express the natural frequency associated with the suspension system.”

The authors apologize for this error and state that this does not change the scientific conclusions of the article in any way. The original article has been updated accordingly.

